# Clinical Hypnosis and Cognitive Behavioral Therapy for Hot Flashes: A Scoping Review

**DOI:** 10.1089/whr.2024.0144

**Published:** 2025-01-08

**Authors:** Vanessa Muñiz, Victor Julian Padilla, Cameron T. Alldredge, Gary Elkins

**Affiliations:** Department of Psychology & Neuroscience, Baylor University, Waco, Texas, USA.

**Keywords:** cognitive behavioral therapy, hot flashes, hypnosis, menopausal, scoping review

## Abstract

**Background:**

Hot flashes can be a prevalent issue for postmenopausal women, but traditional treatments such as hormone therapy can have adverse side effects. Recommended psychotherapies for managing hot flashes include cognitive behavioral therapy (CBT) and clinical hypnosis, but an in-depth review comparing the efficacy of both treatments is warranted.

**Objectives:**

The aim of the present scoping review was to assess the clinical significance and efficacy of symptom reduction of CBT and clinical hypnosis as treatments of hot flashes.

**Eligibility Criteria:**

Peer-reviewed primary studies were included in this review if they were published in English, used CBT or clinical hypnosis as their intervention, included hot flash outcomes, and sampled women aged 18 years or older.

**Sources of Evidence:**

A search was conducted on PubMed, Web of Science, and PsycINFO over December 2023 and January 2024.

**Charting Methods:**

Extracted information included eligible studies’ authors, year of publication, country, title, design, participant population, intervention type, control group, primary and secondary outcomes, and key findings.

**Results:**

Of the 1847 studies identified by the literature search, 23 studies were included in this scoping review. While CBT was found to benefit bother and daily interference related to hot flashes, only studies investigating clinical hypnosis found significant reductions in the frequency and severity of hot flashes.

**Conclusions:**

Clinical hypnosis was found to outperform CBT by a large effect in treatment for hot flashes based on the current state of the literature. While both modalities treat psychological distress, only clinical hypnosis demonstrates the ability to reduce the severity and frequency of hot flashes, thus showing clinical significance. Limitations and future directions for research into CBT and clinical hypnosis for hot flashes are discussed.

## Introduction

Hot flashes are characterized as sudden-onset, transient, and spontaneous sensations of heat usually felt on the chest, neck, and face, accompanied by sweating, flushing, heart palpitations, headache, fatigue, anxiety, and chills.^1,2^ These are most prevalent in menopausal women and breast cancer patients. Approximately 80% of menopausal women and 72.8% of postmenopausal breast cancer survivors (6 years after diagnosis) report experiencing hot flashes.^3–5^ Given their symptomology, hot flashes are disruptive and problematic in the daily life activities for most women. Additionally, they have been reported to negatively impact health and quality of life.^6,7^

Traditional treatment for hot flashes is hormone therapy (HT); however, HT might yield severe side effects including thromboembolic events, stroke, and incidence of breast cancer.^8,9^ Reoccurrence or progression of cancer and risk of thromboembolic disease are safety concerns of HT that are more prevalent in cancer patients.^10^ As a result, several evidence-based, nonhormonal treatments have been explored for the treatment of hot flashes. Most notable are cognitive behavioral therapies (CBTs) and clinical hypnosis. Several literature reviews show that these treatments could be feasible, pleasant psychological interventions that benefit women suffering from vasomotor symptoms such as hot flashes or night sweats.^10–12^ Across these reviews, CBT and clinical hypnosis are expressed to be a recommendable intervention for reducing the impact of vasomotor symptoms in menopausal women and breast cancer survivors. Moreover, the 2023 non-HT position statement provided by the North American Menopause Society reported level 1 evidence for CBT and clinical hypnosis.^13^ In the report, a treatment is considered to have level 1 evidence when there is a sufficient amount of consistent scientific evidence. However, the report did not directly address efficacy or clinical significance of CBT and clinical hypnosis in the reduction of hot flashes versus effect on psychological distress related to hot flashes.

Clinical significance of interventions across studies was reported on this review. Unlike statistical significance, clinically significant findings primarily assess the efficacy of a treatment based on its clinical relevancy on its specific target condition (*i.e.,* hot flashes).^14^ In order to achieve clinical significance, the intervention must improve medical care and result in the improvement of individual’s quality of life (*i.e.,* physical function, mental status, and ability to engage in social life).^15^ Based on previous literature, reductions of at least 50% in hot flashes and daily interference must be present for an intervention to be considered clinically significant.^16,17^ This distinction is necessary because studies that do not include occurrence or frequency of hot flashes in their outcome measures might present interventions that are only effective in alleviating distress caused by hot flashes, and not in the actual reduction of hot flashes. Although those studies could yield significant results for coping with hot flashes, that should not be the only outcome considered when assessing clinical significance of treatment modalities.

This scoping review’s primary aim is to assess the clinically significant efficacy of these level 1 psychotherapies for hot flashes, to further inform clinical practice. Additional aims are to provide a synthesis of the primary literature available on CBT and clinical hypnosis as treatment for hot flashes, and to investigate and discuss the interventions’ additional benefits beyond reduction of hot flashes (*e.g.,* perceived bother and daily interference of hot flashes and health-related outcomes such as sleep, quality of life, depression, anxiety, and sexual functioning). By systematically reviewing and synthesizing the results of previous studies into one easy-to-read document, this article will help facilitate the education of future clinicians and researchers on the use of CBT and clinical hypnosis for the treatment of hot flashes.

## Methods

The present scoping review was conducted over December 2023 and January 2024 under the guidance of the Preferred Reporting Items for Systematic Reviews and Meta-Analyses extension for Scoping Reviews (PRISMA-ScR).^18^ Studies were eligible for inclusion in this review if they were published in English in a peer-reviewed, empirical journal, investigated the use of a CBT or hypnosis-based intervention in women aged 18 years or older experiencing hot flashes, and measured hot flashes as primary or secondary outcomes. Studies without a control or comparison group were included in this review. Reviews, meta-analyses, commentaries, and research protocols were excluded. On December 29, 2023, a search was conducted on PubMed, Web of Science, and PsycINFO to identify articles that met the predetermined eligibility criteria. The search strategy used for PubMed is presented in Table 1.

**Table 1.** PubMed Search Strategy

Search	Query
#1	Hot flashes or hot flushes or vasomotor or night sweats or menopaus* symptom*
#2	Hypno* intervention or hypnosis or hypnotic relaxation therapy or hypnotherapy or hypnotherapeutic or CBT or cognitive restructuring or cognitive technique or cognitive behavioral intervention or cognitive intervention or cognitive behavioral or cognitive treatment
#3	Clinical trial or comparative study or randomized controlled trial or controlled clinical trial or intervention or effectiveness or efficacy
#4	#1 and #2 and #3

CBT, cognitive behavioral therapy.

The primary outcome of this review was reduction of hot flashes as measured through frequency, severity, or hot flash scores. Secondary outcomes encompassed bother/interference caused by hot flashes, as well as impact on health-related outcomes and sleep. These outcomes are consistent with the core outcome set recommended by the COMMA (Core Outcomes in Menopause) initiative for randomized controlled trials (RCTs) assessing vasomotor symptoms to provide rigorous empirical findings.^19^ However, provided that included studies were conducted prior to the publication of these recommendations, several studies failed to measure all of the recommended core outcomes. Therefore, additional secondary outcomes most frequently reported in the literature were included in our results and discussion to supplement our holistic understanding of the studies and their intervention’s impact on management of hot flashes.

The studies identified by this search strategy were reviewed for duplication and screened for eligibility using Covidence (covidence.org). Two reviewers (V.M. and V.J.P.) independently screened the titles and abstracts of studies before moving on to reviewing the full-text of relevant studies. Conflicts at each stage of this screening process were discussed between the reviewers until a consensus was made on whether to include or exclude identified studies into the next stage of screening. The authors, year of publication, country of study, title, study design, participant population, intervention type, control group, primary and secondary outcomes, and key findings of eligible studies were extracted for further analysis and discussion.

## Results

### Study selection

**FIG. 1.**
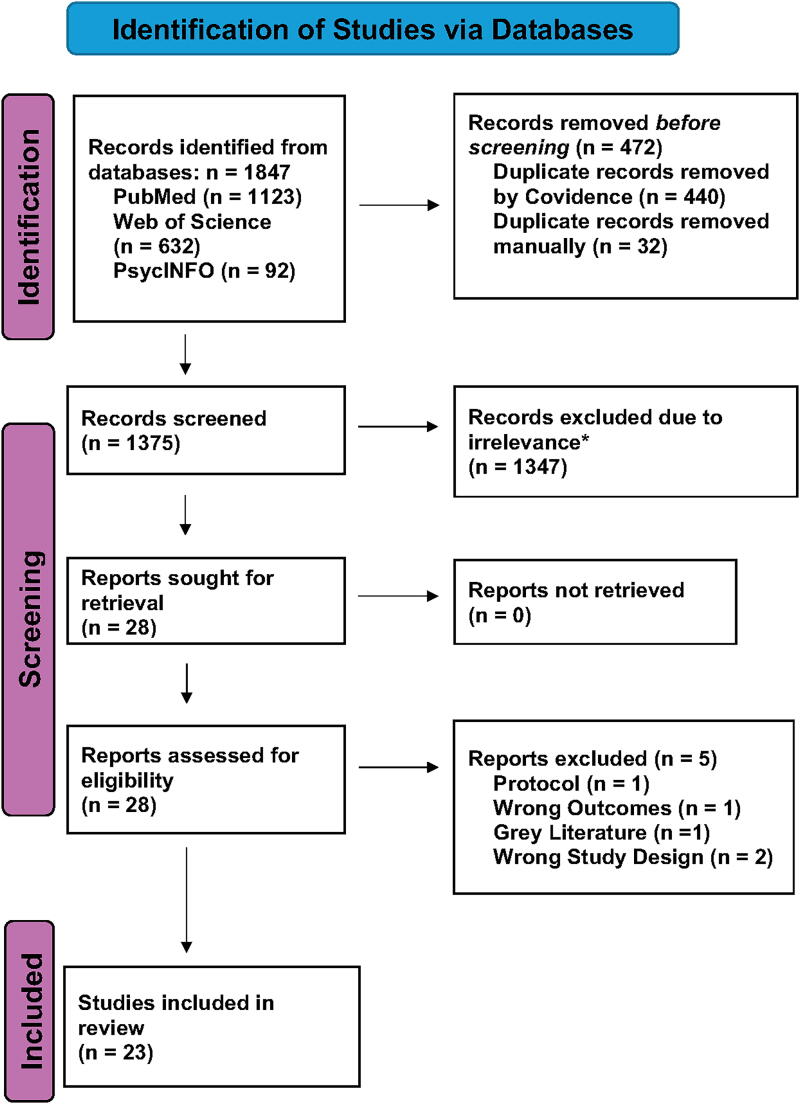
PRISMA flowchart. Note: Adapted from the updated PRISMA 2020 guidelines for reporting systematic reviews.^20^ *Exclusion due to irrelevance included not assessing CBT or clinical hypnosis, no mentioning of hot flashes, stating it was a review article, commentary, book chapter, conference abstract, study protocol, or treatment guide/script. CBT, cognitive behavioral therapy; PRISMA, Preferred Reporting Items for Systematic Reviews and Meta-Analyses.

A total of 1847 studies were identified by the literature search. A total of 1375 studies remained following duplicate removal. Of these, 1123 studies were identified from PubMed, 632 from Web of Science, and 92 from PsycINFO. Note that 1347 studies were excluded from this review following initial screening of their titles and abstracts. Reasons for exclusion were due to irrelevance, specifically not using CBT or a clinical hypnosis intervention, not mentioning measurement of hot flashes or vasomotor symptoms, and stating that it was a review article, study protocol, position or commentary paper, treatment guide or script, book chapter, or conference abstract. Full-text screening of the remaining 28 studies excluded a further five studies yielding a final sample of 23 studies to be included in this review. A PRISMA flowchart along with reasons for exclusion is provided in Figure 1.

### Identified studies

From the 23 included studies, 9 were conducted in the United States,^21–29^ 7 in the United Kingdom,^30–36^ 3 in the Netherlands,^37–39^ 2 in Canada,^40,41^ and 1 each in Iran^42^ and Belgium.^43^ The most recent study included in this review was published in 2022^42^ whereas the oldest was published in 1996.^34^ Seven studies did not include a control or comparison group.^22–24,26,33,37,41^ These studies provided key findings on the feasibility and acceptability of CBT and clinical hypnosis intervention for hot flash management.

While all of the identified studies included women experiencing hot flashes, there was variation in the participants’ menopausal stage and history of breast cancer. Ten studies recruited participants at peri- or postmenopause, with age ranges of approximately 40/45–60/65 years, excluding those with a history of breast cancer. Eight of those studies used a CBT intervention,^23,29,30,32,34,36,40,42^ and two used clinical hypnosis.^26,27^ Moreover, 11 studies only included women that met the criteria of a history of breast cancer or at-risk. Seven of those studies used a CBT intervention,^22,31,33,35,37–39^ and four used clinical hypnosis.^24,25,28,43^ In most of those studies, participants recruited could be 18 years or older. Only two studies included both postmenopausal and/or breast cancer survivors in their studies.^21,41^ A summary of the characteristics and design of studies included in this review can be found in Table 2.

**Table 2.** Summary of Characteristics and Design of Included Studies

Authors(s), year	Country	Title	Study design	Study population
Berlière et al., 2018^43^	Belgium	The Advantages of Hypnosis Intervention on Breast Cancer Surgery and Adjuvant Therapy	Quasi-experimental	*N* = 300 patients undergoing breast surgery such as a lumpectomy or mastectomy either with or without axillary lymph node dissection or sentinel lymph node biopsy. Inclusion and exclusion criteria not reported.
Atema et al., 2017^37^	Netherlands	An Internet-based Cognitive Behavioral Therapy for Treatment-induced Menopausal Symptoms in Breast Cancer Survivors: Results of a Pilot Study	Feasibility study^a^	*N* = 21. Inclusion criteria: Women with confirmed history of breast cancer and of chemotherapy, oophorectomy, and/or hormone therapy, diagnosed before the 53 years of age. experienced sometimes/often hot flashes, night sweats, and/or vaginal dryness in the past 2 weeks. Exclusion criteria: unable to speak Dutch; serious cognitive or psychiatric disorder; concurrent participation in other studies or rehabilitation therapies.
Elkins, Johnson et al., 2013^26^	USA	A Pilot Investigation of Guided Self-hypnosis in the Treatment of Hot Flashes Among Postmenopausal Women	Pilot study^a^	*N* = 13. Inclusion criteria: Postmenopausal women, experienced at least 50 hot flashes per week or seven hot flashes per day. Exclusion criteria: FSH levels lower than 40; concurrent use of other treatment or therapies for hot flash reduction.
Carpenter et al., 2007^22^	USA	Cognitive-behavioral Intervention for Hot Flashes	Feasibility study^a^	*N* = 40. Inclusion criteria: Women with hot flashes, diagnosis or at high-risk of breast cancer. Exclusion criteria: not reported.
Elkins, Fisher et al., 2013^27^	USA	Clinical Hypnosis in the Treatment of Post-Menopausal Hot Flashes: A Randomized Controlled Trial	RCT	*N* = 187. Inclusion criteria: 18 years of age or older, and had no menstrual period in the past 12 months or no menstrual period in the past 6 months with either a medical history of a FSH levels greater than 40 or a bilateral oophorectomy. Self-reporting at least seven hot flashes per day, or 50 hot flashes per week. Exclusion criteria: non-English speakers, concurrent use of other treatments for hot flashes, history of severe psychopathology such as psychosis, borderline personality disorder, or schizophrenia.
Green et al., 2019^40^	Canada	Cognitive Behavioral Therapy for Menopausal Symptoms (CBT-Meno): A Randomized Controlled Trial	RCT	*N* = 90. Inclusion criteria: In menopausal transition or postmenopausal, and experiencing frequent, distressing vasomotor symptoms with a HFRDIS of 30 or more. Exclusion criteria: severe depression, active suicidal ideation, psychosis, substance use disorder, concurrent psychological treatment, and non-English speakers.
Mann et al., 2012^35^	UK	Cognitive Behavioral Treatment for Women Who Have Menopausal Symptoms After Breast Cancer Treatment (MENOS 1): A Randomised Controlled Trial	RCT	*N* = 96. Inclusion criteria: English-speaking, adult women with ten or more disruptive HF/NS for at least 2 months, history of medical treatments for breast cancer with no comorbid cancers or metastases. Exclusion criteria: unable to attend sessions, and seeking CBT treatment for conditions other than HF/NS.
Sadeghijoola et al., 2022^42^	Iran	Comparing the Effects of Face-to-face Versus Phone Counseling Based on Cognitive-Behavioral Therapy for Vasomotor Symptoms in Postmenopausal Women: A Randomized Controlled Trial	RCT	*N* = 40. Inclusion criteria: 40–65 years of age, 1–5 years postmenopause, a score greater than one on the Kupperman hot flash index, and at least 20 hot flashes per week for at least a month. Exclusion criteria: history of psychological and physical illness, drug abuse, and concurrent use of hot flash medications.
Ayers et al., 2012^30^	UK	Effectiveness of Group and Self-help Cognitive Behavior Therapy in Reducing Problematic Menopausal Hot Flushes and Night Sweats (MENOS 2): A Randomized Controlled Trial	RCT	*N* = 140. Inclusion criteria: peri- or postmenopausal women with at least 10 hot flashes with a score >2 on the Hot Flush Rating Scale in the past month, willing to participate in the study. Exclusion criteria: non-English speakers, a history of breast cancer, and concurrent medical or psychiatric conditions that influence the ability to participate in the study.
Fenlon et al., 2020^31^	UK	Effectiveness of Nurse-led Group CBT for Hot Flushes and Night Sweats in Women with Breast Cancer: Results of the MENOS4 Randomized Controlled Trial	RCT	*N* = 127. Inclusion criteria: women 16 years or older with a diagnosis of breast cancer or DCIS, experiencing at least seven HF/NS per week, and at least a 4 on the HFRS.
Barton et al., 2017^21^	USA	Efficacy of a Biobehavioral Intervention for Hot Flashes: A Randomized Controlled Pilot Study	RCT	*N* = 71. Inclusion criteria: postmenopausal women, reporting at least four bothersome hot flashes per day for at least a month. Exclusion criteria: planning to discontinue any ongoing endocrine therapy during the duration of the study, allergies to venlafaxine, concurrent use of medication for the treatment of hot flashes, and uncontrolled hypertension. Participants also could not have used hypnosis, venlafaxine, or other antidepressants in the past 6 months.
Duijts et al., 2012^39^	Netherlands	Efficacy of Cognitive Behavioral Therapy and Physical Exercise in Alleviating Treatment-Induced Menopausal Symptoms in Patients with Breast Cancer	RCT	*N* = 422. Inclusion criteria: women with a diagnosis of primary breast cancer during premenopausal that received chemotherapy and/or hormonal therapy with no concurrent illnesses. Exclusion criteria: non-Dutch speakers, severe cognitive, psychiatric, or physical conditions, obesity, and concurrent participation in other studies for reduction of menopausal symptoms.
Atema et al., 2019^38^	Netherlands	Efficacy of internet-Based Cognitive Behavioral Therapy for Treatment-Induced Menopausal Symptoms in Breast Cancer Survivors: Results of a Randomized Controlled Trial	RCT	*N* = 254. Inclusion criteria: a histologically confirmed diagnosis of breast cancer before the 50 years of age, history of chemotherapy, oophorectomy, and/or endocrine treatment with no concurrent illness at the time of entering the study. Participants also had to report experiencing hot flashes with a score >2 on the Hot Flush Rating Scale in the past 2 months with at least 10 hot flashes occurring in the last week. Exclusion criteria: prior diagnosis of another cancer, no internet access, non-Dutch speakers, concurrent participation in studies or therapies for menopausal symptoms, and severe cognitive or psychiatric disorders.
Hunter & Liao, 1996^34^	UK	Evaluation of a Four-Session Cognitive-Behavioral Intervention for Menopausal Hot Flashes	Quasi-experimental	*N* = 61 women experiencing at least one hot flash/night sweat per week. Additional inclusion and exclusion criteria were not reported.
Hunter et al., 2009^33^	UK	Evaluation of a Group Cognitive Behavioral Intervention for Women Suffering from Menopausal Symptoms Following Breast Cancer Treatment	Pilot study^a^	*N* = 17. Inclusion criteria: English-speaking breast cancer patients over 18 years of age with no concurrent psychiatric disorders seeking treatment for HF/NS. Exclusion criteria was not reported.
Conklin et al., 2020^23^	USA	Manualized Cognitive Behavioral Group Therapy to Treat Vasomotor Symptoms for Women Diagnosed with Mood Disorders	Feasibility study^a^	*N* = 59. Inclusion criteria: peri- or postmenopausal women between 40 and 65 years of age with a diagnosis of mood disorders and at least one HF/NS daily. Exclusion criteria: specific racial groups, severe psychiatric disorders (*i.e.,* borderline personality disorder, psychosis, active suicidal ideology, or substance use disorder), currently taking tamoxifen, chemotherapy, or hormone therapy.
Younus et al., 2003^41^	Canada	Mind Control of Menopause	Pilot study^a^	*N* = 14. Inclusion criteria: women reporting at least 5 hot flashes per week for at least a month. Breast cancer patients were eligible after completion of cancer therapy at least 3 months before starting the study. Exclusion criteria: patients with metastatic breast cancer, concurrent physical or cognitive impairment that impact ability to participate in the study.
Elkins et al., 2007^24^	USA	Pilot Evaluation of Hypnosis for the Treatment of Hot Flashes in Breast Cancer Survivors	Pilot study^a^	*N* = 16 breast cancer survivors experiencing hot flashes. Participants taking venlafaxine were included in the study if it was reported to be ineffective after at least 4 weeks of use. Additional inclusion or exclusion criteria were not reported.
MacLaughlan et al., 2013^28^	USA	Randomised Controlled Trial Comparing Hypnotherapy Versus Gabapentin for the Treatment of Hot Flashes in Breast Cancer Survivors: A Pilot Study	Prospective randomized trial	*N* = 17. Inclusion criteria: women over 18 years of age at high risk or with a history of breast cancer, at least one daily hot flash, and no current use of gabapentin or hypnotherapy. Exclusion criteria: currently undergoing chemotherapy or radiation for breast cancer, and severe psychiatric or clinical illness that might impact ability to participate in the study
Elkins et al., 2008^25^	USA	Randomized Trial of a Hypnosis Intervention for Treatment of Hot Flashes Among Breast Cancer Survivors	RCT	*N* = 60. Inclusion criteria: women18 years or older with a history of primary breast cancer, self-reporting at least 14 weekly hot flashes for at least a month. Exclusion criteria: currently receiving chemotherapy, hormone therapy, or any treatment of hot flashes. Participants taking antihormonal agents for breast cancer for over a month were included if intake remained stable throughout the study.
Hardy et al., 2018^32^	UK	Self-Help Cognitive Behavior Therapy for Working Women with Problematic Hot Flushes and Night Sweats (MENOS@Work): A Multi-Center Randomized Controlled Trial	RCT	*N* = 124. Inclusion Criteria: postmenopausal women, employed from a participating organization, 45–60 years of age, English speaking, reporting a minimum of 10 hot flashes with a score >2 on the Hot Flush Rating Scale for at least two months. Exclusion criteria: severe physical or psychological problems that might impact ability to participate in the study.
McCurry et al., 2016^29^	USA	Telephone Delivered Cognitive-Behavior Therapy for Insomnia in Midlife Women with Vasomotor Symptoms: An MsFLASH Randomized Trial	RCT	*N* = 106. Inclusion criteria: peri- or postmenopausal women, 40–65 years of age, with insomnia and at least two daily hot flashes for 2 weeks. Exclusion criteria: diagnosis of a primary sleep disorder, consumption of more than three daily alcoholic drinks, or an illness that severely impacts sleep, continuous use of prescribed sleep medication, or current employment in shift work.
Stefanopoulou and Hunter, 2014^44^	UK	Telephone-Guided Self-Help Cognitive Behavioral Therapy for Menopausal Symptoms	Quasi-experimental	*N* = 47. Inclusion criteria: peri- or postmenopausal women, English-speaking, reporting at least 10 hot flashes with a score >2 on the Hot Flush Rating Scale for a month, unable to attend face-to-face interviews. Exclusion criteria: non-English speakers, with a history of psychiatric or medical conditions that might impact the ability to participate in the study.

^a^ Study does not include a control or comparison group.

CBT, cognitive behavioral therapy; HFRDIS, Hot Flash Related Daily Interference Scale; FSH, Follicle-stimulating hormone; DCIS, Ductal carcinoma in situ; HFRS, Hot Flush Rating Scale.

### Interventions observed

The studies varied in the delivery of the CBT or hypnosis interventions. Table 3 provides a more descriptive outline of each study’s intervention. From the overall included studies, eight reported administering clinical hypnosis as their intervention,^21,24–28,41,43^ and 15 studies delivered CBT.^22,23,29–40,42^ Six CBT trials were delivered remotely, providing participants with either internet-based,^38^ prerecorded DVD,^22^ self-help booklets,^32,36^ or telephone-based interventions.^29,42^ One study compared the effectiveness of group CBT to self-help CBT^30^ and another study compared in-person CBT to physical exercise.^39^ One of the hypnosis studies relied primarily on self-hypnosis^26^ and seven out of the eight studies using clinical hypnosis reported in-person hypnosis sessions, where participants met with a trained clinician for the duration of the treatment.^21,24,25,27,28,41,43^ Five of these studies included self-hypnosis training in their treatment plan, to provide participants with tools for hot flash management they can practice at home.^24,25,27,28,43^

**Table 3.** Intervention and Comparator Outcomes of Studies Using CBT or Clinical Hypnosis for the Reduction of Hot Flashes

Study	Intervention	Control	Primary outcome	Secondary outcome	Key findings
Berlière et al. (2018)^43^	Clinical hypnosis was delivered before, during and after the operation by an anesthesiologist. Suggestions included experiencing positive dreams and memories, comfort, and healing.	General anesthesia	Surgery duration, hospitalization duration, asthenia severity, anxiety symptom severity, incidence of radiodermitis, incidence of nausea, incidence of hot flashes, incidence of joint and muscle pain, endocrine therapy compliance: Self-report questionnaires and interviews; lymph production, postmastectomy lymph punctures: lymph node biopsy and dissection.	No secondary outcomes were reported.	Hypnosis sedation significantly reduced: frequency of severe hot flashes (*p* < 0.001), joint or muscle pain (*p* < 0.001), and asthenia (*p* < 0.001). Clinical hypnosis as a sedative was effective in reducing duration of hospitalization from 4.1 to 3 days (*p* < .001) for all surgical procedures, lymph punctures postmastectomy and anxiety.
Atema et al. (2017)^37^	Participants received weekly, one-hour long CBT modules spread throughout six weeks. Module components included psycho-education on breast cancer and menopause, hot flashes, stress and relaxation, sleep, body image/ sexuality, reflection, and maintenance plan.	No control group	Program usage and evaluation of intervention: Self-reported questionnaires and interviews with counselors.	Overall levels of endocrine symptoms: Functional Assessment of Cancer Therapy Questionnaire. Hot flash problem ratings: Hot Flush Rating Scale	Participants reported high percentages of satisfaction (88.8%), user-friendliness (94.4%), and 19 out of 21 of the participants completed the 6-week program. Internet-based CBT program significantly improved self-reported bother/daily interference of hot flashes in 61% and endocrine levels in 72% of the participants. Frequency of hot flashes were not assessed.
Elkins, Johnson et al. (2013)^26,^^a^	Participant received five weekly, 30-minute sessions of guided self-hypnosis *via* audio recording. The intervention included suggestions for mental imagery of coolness, relaxation, symptom reduction, and guidance for future self-hypnosis without audio.	No control group	Hot flash frequency and severity rating: Hot Flash Symptoms Diary	No secondary outcomes were reported	Self-guided hypnosis significantly lowered hot flash frequency by about 72% and severity ratings by 76% after treatment.
Carpenter et al. (2007)^22^	Participants watched a DVD of CBT and were instructed to practice the techniques taught in the video for 1 week. Components included video clips on distraction as a cognitive activity, guidance to remain still, and a breathing exercise.	No control group	Acceptability of the mode of delivery Physiological hot flashes: a skin conductance monitor. Hot flash severity and bother: Self-reported numeric scales	Mood disturbance: Center for Epidemiological Studies — Depression scale; Profile of Mood States — Short Form. Hot flash disturbance: Hot Flash Related Daily Interference Scale. Sleep disturbance: A wrist actiwatch.	There were no significant changes reported in physiological hot flashes. Participants reported about a 10% decrease in hot flash severity, bother, and HFRDIS scores
Elkins, Fisher et al. (2013)^27^^,a^	The intervention consisted of five 45-minute sessions of hypnotic inductions with guidance for self-hypnosis toward the therapeutic goal. The intervention included personalized suggestions for mental imagery of coolness, safe place, and relaxation.	Structured- attention control	Physiological hot flashes: a skin conductance monitor Hot flash frequency: Hot Flash Symptoms Diaries.	Hot flash disturbance: Hot Flash Related Daily. Interference Scale Sleep quality: Pittsburg Sleep Quality Index.	Clinical hypnosis significantly reduced hot flash frequency (63.87%), score (71.36%), daily interference (69.02%), sleep quality (43.49%), and physiologically recorded hot flashes (40.92%). These results stayed consistent at their 12-week follow-up.
Green et al. (2019)^40^	Consisting of 12 weekly CBT sessions. Sessions were two hours in length in groups of eight participants. Contents included psychoeducation, conceptualization and behavioral strategies for vasomotor symptom monitoring, depression, sleep, anxiety, urogenital complaints, and sexual concerns.	Waitlist control	Vasomotor symptoms: Hot Flash Related Daily Interference Scale Depression: Beck Depression Inventory (BDI-II)	Symptom severity: vasomotor subscale of the Greene Climacteric Scale; the Montgomery-Asberg Depression Rating Scale Anxiety: Hamilton Anxiety Scale (HAM-A) Sleep Difficulty: Pittsburg Sleep Quality Index Sexual concerns: Female Sexual Function Index; sexual concern item from the Greene Climacteric Scale	CBT intervention group improved daily interference from a score of 56.24 at baseline to 30.54 at week 12. Anxiety, depression, and sleep quality also decreased in the CBT group more than those on the waitlist control. Frequency of hot flashes was not assessed.
Mann et al. (2012)^35^	Group CBT consisted of six weekly sessions of 90 minutes in length each. The sessions were composed of interactive psycho-educations on physiological, cognitive, behavioral, and emotional components of hot flashes.	Usual care	Hot flashes and night sweats problem rating: Hot Flush Rating Scale	Follow-up problem ratings of hot flashes: Hot Flush Rating Scale Hot flash frequency: Sternal skin conductance monitoring and daily diaries Mood: Women’s Health Questionnaire Quality of life: General Health Survey Short Form 36	There were no significant differences between the CBT (21% reduction) and control group (24% reduction) in hot flash frequency using both sternal skin conductance monitoring and daily diaries. Hot flash problem ratings, depression, sleep disturbances and anxiety and were lower in the CBT group; however, differences in anxiety and somatic symptoms were not statistically significant.
Sadeghijoola et al. (2022)^42^	The phone-delivered CBT consisted of six weekly sessions of 30–40 minutes in length. The first and fourth sessions had face-to-face components, and counseling included breathing and relaxation techniques.	In-person CBT	Hot flash and night sweats symptoms were measured using a diary.	No secondary outcomes were reported.	Face-to-face CBT had hot flash frequency reductions from 31.92 at baseline to 18.83 at endpoint, severity from 2.24 to 1.21, and duration from 4.22 to 2.79. Phone CBT had hot flash frequency reductions from 33.32 to 19.53, severity from 2.23 to 1.20, and duration from 4.29 to 2.77. There were significant within-group differences (*p* < 0.001) but no significant between-group differences in-person and phone-delivered CBT groups across all variables.
Ayers et al. (2012)^30^	Group CBT was administered in four weekly 2-hour sessions. Components included psychoeducation, strategies for dealing with hot flashes such as stress management and paced breathing, night sweats and sleep strategies.	No treatment control group and self-help CBT	Hot flash and night sweats problem ratings: Hot Flush Rating Scale	Follow-up problem ratings of hot flashes at week 26: Hot Flush Rating Scale Physiologically measured hot flash frequency: Sternal skin conductance monitoring Mood, sleep: Women’s Health Questionnaire Quality of life: General Health Survey Short Form-36	There were no significant differences in hot flash frequency between the three groups for both physiologically nor self-reported. Group CBT reduced hot flash frequency by 40%, self-help CBT by 36% and no treatment control by 23%. Group CBT improved mood and quality of life better than self-help CBT. Both CBT groups had significantly less bother/daily interference than the control group.
Fenlon et al. (2020)^31^	CBT consisted of six weekly 90 minute sessions with components including psycho-education and strategies for stress and hot flash management, improve well-being, paced breathing, and sleep.	Usual care	Hot flash problem ratings: HFNS Problem Rating Scale. Hot flash frequency: 3-day diary card	Follow-up problem ratings of hot flashes at week 9 and 26 and hot flash frequency: Hot Flush Rating Scale Beliefs about hot flashes: the Short Form Hot Flush Beliefs and Behaviors Scale Quality of life: Hot Flash Related Daily Interference Scale	There was a 46% reduction in daily interference due to hot flashes and night sweats and a 28% reduction in hot flash frequency in the CBT intervention group. There were also improvements in sleep quality, anxiety, and depression scores at 9 and 26 weeks posttreatment.
Barton et al. (2017)^21^^,a^	Women were randomized to receive venlafaxine 75 mg or a placebo, and after a week, they received four weekly, 20-minute sessions of either hypnosis or sham hypnosis. In the hypnosis intervention group, suggestions included deep relaxation, feelings of safety, reduced anxiety, and suggestions for coolness.	Placebo medication and sham hypnosis	Hot flash score: daily diary log Side-effects assessment: questionnaire	Quality of life: Menopausal Quality of Life Perception of change: Subject Global Impression of Change	All active treatment groups had a reduction of hot flash frequency over 50%. Women in the venlafaxine/hypnosis reported hot flash reductions similar to women in the placebo/hypnosis group and venlafaxine/sham hypnosis group. These three groups performed better than the placebo/sham hypnosis group.
Duijts et al. (2012)^39^	CBT, physical exercise, and a combination of both were the interventions observed. The CBT program consisted of six weekly 90-minute sessions focusing on relaxation exercises.	Waitlist control	Endocrine symptoms: Functional Assessment of Cancer Therapy Questionnaire — Endocrine Subscale Hot flashes frequency and problem ratings: Hot Flush Rating Scale	Sexual functioning: Sexual Activity Questionnaire Urinary symptoms: Bristol Female Lower Urinary Tract Symptoms Questionnaire Body image: European Organization for Research and Treatment of Cancer Quality of Life Breast Cancer Questionnaire Psychological distress: Hospital Anxiety and Depression Scale Quality of life: 36-Item Short Form Health Survey	There were significant improvements in the CBT groups on hot flash bother and daily interference (*p* < 0.001) and short-term endocrine symptoms (*p* < 0.001). However, there were no significant differences in hot flash frequency.
Atema et al. (2019)^38^	Therapist and self-guided internet-based CBT interventions were composed of six weekly 1-hour sessions. Each session contained psychoeducation, self-reflection, and strategies for managing hot flashes, stress management, and sleep.	Waitlist control	Hot flash problem ratings: Hot Flush Rating Scale Menopausal symptoms: Functional Assessment of Cancer Treatment — Endocrine Symptoms	Sleep quality: Groningen Sleep Quality Scale Frequency of hot flashes: Hot Flush Rating Scale — frequency subscale Sexual functioning: Sexual Activity Questionnaire Psychological distress: Hospital Anxiety and Depression Scale Quality of life: 36-Item Short Form Health Survey	In the CBT guided group, overall menopausal symptoms decreased by 44.4% (compared with 22% in the control group) and 45.6% of participants self-reported clinically significant improvements in daily interference due to hot flashes (compared with 26.3% in the control group). In the internet-based CBT group, overall menopausal symptoms decreased by 39 % and 39% of participants reported clinically significant lower perceived impact of hot flashes.
Hunter & Liao (1996)^34^	The CBT intervention consisted of four hourly sessions spread throughout 6–8 weeks. The sessions included psychoeducation on menopause and stress, and cognitive-behavioral strategies to improve relaxation and reduce panic and stress.	Waitlist control and HT	Hot flashes: daily diaries, Problem Rating Scale	Women’s Health Questionnaire, and self-esteem scales	CBT improved self-reported hot flash frequency and bother by about 50%, as well as anxiety and depression.
Hunter et al. (2009)^33^	Group CBT was delivered in six 1.5-hour long sessions. It included psycho-education on hot flashes, night sweats, and menopause, as well as strategies for relaxation, stress reduction, improve sleep, and monitor precipitants.	No control group	Hot Flash Frequency and Problem Rating Scale	Mood and sleep: Women’s Health Questionnaire Quality of life: 36-Item Short Form Beliefs about menopausal symptoms: Hot Flush Beliefs Scale	There was a 38% reduction in hot flash frequency and a 49%–59% reduction in bother/daily interference as measured by problem ratings. Anxiety and depression symptoms were reduced significantly.
Conklin et al. (2020)^23^	The group CBT was delivered in six weekly 90-minute sessions in groups of four–seven participants. The sessions were audio-delivered interventions on the psychoeducation of stress, hot flashes, and relaxation. These also included guidance on relaxation, paced breathing, and deep muscle exercises.	No control group	Hot flash problem rating: Hot Flush Rating Scale, Hot Flash Related Daily Interference Scale	Hot flash frequency: Hot Flush Rating Scale, Hot Flush Daily Diary Mood: Snaith-Hamilton Pleasure Scale, Montgomery-Asberg Depression Rating Scale, Structured Interview Guide for the Hamilton Anxiety Rating Scale, Quick Inventory of Depression Symptomatology — Self Report, Perceived Stress Scale Cognitive appraisal of menopause: Menopause Representations Questionnaire, Hot Flushes Beliefs Scale	The majority of the participants reported improvements in hot flash related daily interference/problem ratings (39.3% reduction), anxiety, and quality of life. There were no significant changes in hot flash frequency and depression scores on the Montgomery-Asberg rating scale.
Younus et al. (2003)^41^^,a^	The hypnotic intervention was delivered in groups of four–five participants spread throughout four weekly sessions. It included suggestions for relaxation, and hot flash reduction and blocking.	No control group	Frequency, duration, and severity of hot flashes: hot flash diary	Quality of life, insomnia and overall health: QLQ-C30 Fatigue: Brief Fatigue Inventory Form	The frequency, duration, and severity of hot flashes were significantly reduced during and after the hypnotic interventions. Overall quality of life and insomnia were also significantly reduced during the whole intervention and fatigue improved during the first three weeks of treatment.
Elkins et al. (2007)^24^^,a^	The clinical hypnosis intervention was comprised of four weekly 45-minutes sessions with suggestions for relaxation and mental imagery for coolness.	No control group	Hot flash scores were measured by multiplying daily frequency by average severity of hot flashes from a daily diary	Hot flash interference: Hot Flash Related Daily Interference Scale	Hot flash scores had a 70% reduction. Improvements in the daily interference of the hot flashes was also statistically significant.
MacLaughlan et al. (2013)^28^^,a^	Clinical hypnosis was delivered throughout three weekly 1-hour sessions composed of suggestions for relaxation, gaining control over menopausal symptoms, and personalized mental imagery for coolness.	Gabapentin 900 mg daily	Frequency and severity of hot flashes: Daily hot flash diary	Quality of life: Hot Flash Related Daily Interference Scale	Daily hot flashes reduced by 80% in the group receiving clinical hypnosis and 33.3% in the group receiving gabapentin. Severity of hot flashes also reduced significantly by the hypnosis group (85%) compared with the control group (33.3%). Reduction of daily interference was stable across both groups (55.2% and 51.6%, respectively).
Elkins et al. (2008)^25^^,a^	The clinical hypnosis intervention had five 50-minute weekly sessions with personalized suggestions for mental imagery for coolness, relaxation, and positive imagery of the future.	Waitlist control	Hot flash frequency and severity: daily diary	Interference of hot flashes: Hot Flash Related Daily Interference Scale Sleep: Medical Outcomes Study Sleep Scale Mood: Hospital Anxiety and Depression Scale-Anxiety Subscale, Center for Epidemiologic Studies Depression Scale	The clinical hypnosis intervention group had statistically and clinically significant improvements in hot flash scores (frequency and severity reduced by 68%), and daily interference (*p* < 0.001). There were also statistically significant improvements in depression, anxiety, sleep, and quality of life.
Hardy et al. (2018)^32^	The self-help CBT was delivered in the form of a booklet with four chapters to be completed over 4 weeks. Chapters included topics on psychoeducation about menopause and hot flashes, stress management, breathing exercises, and strategies for the management of hot flashes, stress reduction, and improve sleep.	Waitlist control	Hot flash problem ratings: Hot Flush Rating Scale	Hot flash frequency: Hot Flush Rating Scale Work and social adjustments: Work and Social Adjustment Scale, Stanford Presenteeism Scale Sleep: Pittsburgh Sleep Quality Index Mood: Revised Women’s Health Questionnaire Hot flashes and night sweats beliefs and behaviors: Hot Flush Belief Scale, Hot Flush Behavior Scale, Menopause Representations Questionnaire	Participants in the self-help CBT intervention group reported hot flash problem ratings, levels of work and social adjusting, and sleep were improved. Frequency was reduced by 24% and 35.5% at the 6th and 20th week follow-ups in the self-help CBT group.
McCurry et al. (2016)^29^	The intervention was comprised of six 20–30-minute telephone-delivered CBT sessions completed over 8 weeks. The sessions focused on psycho-education and strategies for improving sleep and stimulus control.	Menopause education control	Sleep quality: Insomnia Severity Index, Pittsburg Sleep Quality Index	Hot flashes and night sweats: Daily sleep diaries, Hot Flash Related Daily Interference Scale Treatment Satisfaction: Self-reported questionnaire	CBT significantly improved daily interference (*p* = 0.03), insomnia (*p* < 0.001), and sleep quality (*p* < 0.001) improved in the group receiving CBT. There were no significant differences in hot flash frequency, severity, or bother between the CBT and control group at weeks 8 or 24.
Stefanopoulou and Hunter (2014)^44^	The self-help CBT intervention was delivered *via* a booklet and was expected to be completed over 4 weeks. Content included psycho-education, paced breathing, relaxation, and techniques for the management of hot flashes.	No control group	Hot flash problem rating: Hot Flush Rating Scale	Hot flash frequency: Hot Flush Rating Scale Beliefs and behaviors: Hot Flush Beliefs Scale, Hot Flush Behavior Scale Sleep, anxiety, and depression: Women’s Health Questionnaire	The group in the self-help CBT self-reported reductions in problem ratings of about 40% and in frequency of hot flashes. Participants also reported a significant increase in mood, coping, and sleep.

^a^ The intervention’s efficacy in treating hot flashes was clinically significant.

HT, hormone therapy.

### Measurements for hot flashes

Diverse outcome measures were used to assess frequency and bother/daily interference of hot flashes. Those used for assessing the frequency and severity of hot flashes included skin conductance monitors for recording hot flashes physiologically^22,27,30,35^ and daily diaries.^21,23–31,33–35,37,38,41,42^ Despite being considered the gold standard for measuring hot flashes in menopausal research,^45^ only four studies recorded hot flashes physiologically. This is probably due to several limitations of skin conductance monitors, including an inability to document hot flashes/ night sweats (HF/NS) severity and low concordance to self-reported hot flash frequency across scales and hot flash diaries outside of a controlled lab setting.^44–46^ Daily hot flash diaries^16^ are self-reported measures in which participants record daily occurrences of hot flashes at onset and categorize them based on severity. In most studies, patients are asked to document their hot flashes every day for 7 days, usually at baseline and for the number of weeks that they are undergoing treatment. Hot flash severity is divided in four different categories (mild, moderate, severe, and very severe). And across several studies, an overall hot flash score can be calculated by multiplying the frequency of hot flashes by the average hot flash severity.

Additional measurements used were to obtain the participants’ perceptions of daily interference or bother caused by hot flashes. These included the Hot Flash Related Daily Interference Scale (HFRDIS),^22–25,27–29,31,40^ the Hot Flush Rating Scale (HFRS),^23,30–39^ the Vasomotor subscale of the Greene Climacteric Scale,^40,47^ and the Work and Social Adjustment Scale.^32,48^ The HFRDIS^49^ is a 10-item scale measuring the degree to which hot flashes interfere with nine everyday activities specific to the impact of hot flashes and overall quality of life. The HFRS^50^ is also a self-reported measure in which participants identify across three Likert scales the extent in which their hot flash symptoms are problematic, distressing, or cause interference in daily life. The HFRS is highly correlated to the HFRDIS (*r* = 0.74, *p* < 0.001), but has a low correlation to frequency of hot flashes (*r* = 0.22–0.39).^51^ Therefore, these assessments of bother/interference aim to measure a different dimension of hot flashes other than hot flash frequency.

### Reduction of hot flash frequency

Clinical hypnosis was successful in providing clinically significant improvements in physiologically and/or self-reported hot flash frequency across all studies. Participants in the hypnosis intervention arm in MacLaughlan and colleagues’ trial^28^ reported an 80% and 85% decrease in hot flash frequency and severity, respectively, whereas the control group receiving gabapentin only reported a 33.3% reduction in both. In a different study comparing hypnosis with venlafaxine for hot flash treatment, both treatment groups (venlafaxine only and hypnosis only) had statistically significant reductions of over 50%, compared with 25% in the double placebo group.^21^ In a single-arm pre–post intervention study for postmenopausal women, clinical hypnosis yielded a mean reduction of 72% in hot flash frequency and 76% in scores.^26^ A randomized clinical trial for hot flashes in breast cancer survivors found a 68% decrease in scores^25^ and participants in a previous pilot study reported a 59% reduction in hot flash frequency and 70% improvement in hot flash scores after the hypnosis intervention.^24^ Finally, Elkins and colleagues reported a reduction in hot flash frequency and scores of 63% postintervention and 74% at 12-week follow-up, compared with reductions of 9% postintervention and 15%–17% at follow-ups in their control groups.^27^

A nonrandomized clinical pre–post trial on sedative hypnotic intervention for breast cancer surgery also had a statistically significant improvement in frequency and severity of hot flashes in the group receiving hypnosis (*p* < 0.001).^43^ However, further information is needed on the measures used for primary outcomes.

On the contrary, studies of CBT interventions for the reduction of hot flashes had mixed findings in hot flash frequency. In Hunter and Liao’s RCT,^34^ hot flush frequency was reduced by about 50% in the groups receiving CBT. The CBT intervention group had a mean decrease in hot flashes from 28 hot flashes at baseline to 14 at intervention, whereas the HT intervention group had a decrease from 42.92 at baseline to 11.75 average hot flashes. However, these findings were not consistent in later studies. Six studies did not find any statistically significant improvements in physiologically and/or self-reported hot flash frequencies,^22,23,29,30,35,39^ two studies did not measure hot flash frequency at all,^37,40^ and seven studies reported small to moderate reductions in frequency of hot flashes.^31–34,36,38,42^ In those that found some reductions, the results in the CBT trials were smaller than those found in clinical hypnosis interventions and were not considered clinically significant because they failed to report over 50% of hot flash reduction.

In a single group trial, patients reported a 38% reduction in hot flash frequency following treatment.^33^ Among RCT studies reporting improvements in hot flash frequency, two studies compared telephone-guided to in-person CBT.^36,42^ In the most recent study, weekly average hot flashes decreased from 31.92 to 18.83 in the in-person CBT group and from 33.32 to 19.53 in the phone counseling group.^42^ In the second study, hot flash frequency as reported in the HFRS decreased from 55 hot flashes at baseline to 37 hot flashes per week in the telephone-guided treatment.^36^ These results were compared with the findings of a previous RCT, which did not find statistically significant reduction of hot flashes compared with the control group.^30^ Other RCTs reported reductions of 35.5% after self-help CBT compared with 15% in the control group at week 20,^32^ and 28% in participants receiving CBT compared with 11% in the control group at week 26,^31^ and overall menopausal symptoms decreased by 44.4% in an internet-based CBT group compared with 22% in the waitlist control group.^38^ Finally, in Hunter and Liao^34^ hot flush frequency was reduced by about 50% in both the CBT group and the CBT waitlist group alike. The CBT intervention group had a mean decrease in hot flashes from 28 hot flashes at baseline to 14 at intervention, whereas the HT intervention group had a decrease from 42.92 at baseline to 11.75 average hot flashes.

Only four studies assessed hot flashes physiologically using skin conductance monitors in addition to the measures aforementioned.^22,27,30,35^ These studies found that CBT did not reduce hot flash frequency, and clinical hypnosis yielded clinically significant reductions in physiologically measured hot flash frequency. In a secondary analysis study combining the MENOS1^35^ and MENOS2^30^ trial studies, CBT had a small, statistically significant reduction of physiologically measured hot flash frequency, but this was only found in nonbreast cancer participants when using daytime data only.^44^ The clinical hypnosis treatment group in Elkins and colleagues^27^ reported a 63.87% reduction in hot flash frequency (compared with 9.24% in the control group), 40.92% in physiologically recorded hot flashes (7% in the control group), 71.36% improvements in hot flash scores, and 69.02% in daily interference (compared with 8.32% and 18.08% in the control groups, respectively). These results stayed consistent at their 12-week follow-up. This study was the first to note significant, physiologically recorded, reductions of hot flash frequency using a behavioral intervention.

### Management of hot flash bother/daily interference

Only CBT intervention trials used the HFRS’s problem ratings to assess bother caused by hot flashes. Across these trials, the majority of participants reported no improvement in reduction of hot flash frequency, but better subjective perception on bother/daily interference of hot flashes. These findings were stable throughout 3–4-month follow-ups.^33,34,36^ Hypnosis studies saw statistically significant improvements of hot flash interference across all studies using the HFRDIS,^24,25,27^ which were consistent at 12-week follow-up.^27^ In MacLaughlan and colleagues^28^ participants in the hypnosis treatment group saw a 55.2% reduction in HFRDIS scores, which was comparable to the group receiving gabapentin as treatment (51.6%). Four out of the five CBT studies using the HFRDIS reported that hot flash daily interference was significantly decreased posttreatment^23,29,31,40^ and in one study participants saw a small decrease of about 10%.^22^

### Health-related outcomes

Most studies included outcomes to assess health-related outcomes such as sleep, quality of life, and psychological measures. The most common quality of life outcome measure was the 36-Item Short Form Health Survey^,30,33,35,38,39^ and the most common measure for mood disturbances was the Women’s Health Questionnaire.^30,32–36^ Five studies showed improvement of quality of life and mood after clinical hypnosis^21,24,25,41,43^ and eight studies showed improvement after CBT.^23,30,31,33–36,40^

#### Sleep quality

Fifteen studies observed sleep as secondary or primary outcomes.^21,22,24,25,27,29–33,35^,^36^,^38^,^40^,^41^ The Pittsburg Sleep Quality Index^52^ was the most frequently used questionnaire,^22,27,29,32,40^ and other measures used for sleep included subscales from the HFRDIS and the Women’s Health Questionnaire.^53^ Only one study used a wrist actigraphy watch to measure sleep disturbance, which found that DVD-delivered CBT did not improve sleep disturbance.^22^ From these studies assessing sleep, 9 out of 10 CBT intervention studies^29–33^,^35^,^36^,^38^,^40^ and all 5 clinical hypnosis intervention trials^21,24,25,27,41^ saw improvements in sleep quality.

#### Sexual functioning

Three studies investigating the effects of CBT included sexual functioning as a secondary measure.^38–40^ Green and colleagues found that postmenopausal women given CBT for menopausal symptoms reported a significantly greater reduction in sexual concerns on the Greene Climacteric Scale at 12 weeks postbaseline than those in the waitlist control condition. There were no significant changes in sexual functioning as measured on the Female Sexual Function Index.^54^ In their 2019 study testing the efficacy of an internet-based CBT intervention for menopausal symptoms in breast cancer survivors, Atema and colleagues did not observe any significant changes in sexual functioning using the Sexual Activity Questionnaire (SAQ).^55^ Finally, a 2012 study by Duijtis found that when combined with physical exercise CBT did significantly improve SAQ scores relative to a waitlist control at 6-month follow-up but not at earlier time points.

#### Anxiety and depression

Ten studies included anxiety or depression as secondary outcomes.^22,23,31,33–37,40,43^ Two studies did not find that CBT for menopausal symptoms was associated with a significant change in anxiety or depression symptoms.^37,40^ Significant reductions in anxiety and depressive symptoms following CBT and clinical hypnosis interventions were found across several studies.^25,31,33–36,43^ However, several CBT trials reported that reductions in depressive symptoms persisted in these studies at follow-up several months after completion of the intervention, while those of anxiety did not.^33–35^ Carpenter and colleagues^22^ noted significant reductions in depressive symptoms only in a subset of participants with the worst hot flash symptoms, and Conklin et al.^23^ reported significant reductions in anxiety but not depressive symptoms compared with baseline.

### Mediators and moderators

In addition to the effectiveness of the intervention, several studies considered potential moderating/mediating variables of the primary outcome in follow-up studies. In a secondary analysis study for potential mediators and moderators of CBT’s effect on hot flash problem ratings reported in the MENOS1 trial, the intervention was effective in reducing hot flash problem ratings regardless of age, BMI, history of breast cancer diagnosis, and type of treatment.^56^ A follow-up study of the MENOS2 trial reported that CBT’s perceived influence on hot flash problem ratings were moderated by completion of the intervention and mediated by changes in cognition such as beliefs about coping and control of hot flashes and beliefs about sleep.^57^ Similarly, CBT’s effect on hot flashes in Atema et al.^38^ was also mediated largely by beliefs about coping and control of hot flashes as well as beliefs on hot flashes in a social context.^58^ Moreover, education level was a significant moderator for this study. Participants reporting a lower education (*i.e.,* completed secondary or vocational education) had greater improvements in perceived impact of hot flashes than those with higher education (completed college/university education).

Hypnotizability, or a person’s ability to respond to hypnotic suggestions,^59^ was found to be a potential moderator of hot flash scores across two RCTs.^25,27^ In Elkins and colleagues’ 2008 trial, participants with higher hypnotizability scores at baseline reported greater improvements than those with lower hypnotizability scores; however, 59 out of 60 participants showed improvements in hot flash scores after clinical hypnosis despite of their hypnotizability scores.^60^ Similarly, in Elkins and colleagues’ 2013 trial, hypnotizability was a significant moderator of hot flash frequency across all assessment points except for participants ranked very lowly hypnotizable.^61^

Moreover, a follow-up study of Elkins and colleagues’ 2013 RCT assessed if levels of salivary cortisol could mediate changes in physiologically, self-reported frequency of hot flashes, as well as hot flash-related daily interferences.^62^ This study found that cortisol levels were significantly decreased in early evening, pre- to postintervention; thus, agreeing with the hypothesis that clinical hypnosis ameliorated stress. However, salivary cortisol changes in pre- to postintervention did not mediate the effects of hypnotic interventions on hot flashes. In another follow-up study, response expectancies and hypnotizability were analyzed to further examine possible mediators of outcomes.^61^ Results found that response expectancy did not mediate hot flash frequency regardless of hypnotizability. Therefore, the effects of clinical hypnosis on hot flash frequency reduction were not mediated by an expectancy or placebo effect.

## Discussion

The primary aim of the present review was to synthesize and evaluate the effectiveness of CBT and clinical hypnosis in managing hot flashes, which are the only nonpharmacological treatments recommended with level 1 status (good and consistent scientific evidence) by the North American Menopause Society.^13^ To accomplish this, we synthesized findings from 23 studies spanning from 1996 to 2022, with varied geographical distributions and methodologies, providing a comprehensive overview of the field. We found that both interventions yielded ancillary benefits such as improved psychological well-being, which is often compromised in individuals experiencing hot flashes. However, this scoping review reveals several important discrepancies between the outcomes of the interventions.

The majority of studies examining the efficacy of CBT interventions for the treatment of hot flashes focused on participants’ daily interference or bother due to hot flashes, two outcome variables strongly correlated with patients’ help-seeking and quality of life. These results were generally favorable in most studies; however, these findings were not correlated with hot flash frequency and therefore not effective in the direct reduction of hot flashes.^50,63^ Studies investigating the use of CBT to treat hot flashes outlined small or null findings on self-reported and physiologically reported hot flash frequencies. Among the included studies, CBT was consistently found to be useful in reducing hot flashes’ daily interference through cognitive restructuring, and not in reducing the occurrence and frequency of hot flashes.

On the contrary, our analysis revealed that trials using clinical hypnosis consistently had clinically significant efficacy to treat and reduce hot flashes directly. Specifically, research on clinical hypnosis reported significant reductions in hot flash frequency and severity across all RCTs^21,25,27,28^ and single-arm trials.^24,26,41^ These findings place hypnosis as the first behavioral intervention to date to report significant improvements in physiologically reported hot flashes.^27^ Studies found that clinical hypnosis was significantly correlated with improved quality of life, sleep quality, and mood with few, if any, adverse events. Notably, reductions of hot flash frequency through hypnosis interventions even surpassed the reductions observed in the control groups receiving either gabapentin or venlafaxine.^21,28^

Although clinical hypnosis has demonstrated clinically significant efficacy in reducing the frequency and severity of hot flashes, the mechanisms remain largely unknown. However, some speculative theories can be formed. Past studies have demonstrated that the effect of hypnotherapy in reducing hot flashes is not attributable to response expectancy or placebo.^61^ Because of this, alternative theories for the mechanisms of hypnosis must be considered. The physiological mechanisms underlying hot flashes involve complex interactions between thermoregulatory control, hormonal changes, and activity in the central nervous system. More specifically, hot flashes are thought to be initiated within the medial preoptic area of the hypothalamus, activating heat-loss mechanisms at normal core body temperatures.^63^ Postmenopausal hot flashes are not solely explained by estrogen withdrawal, as past research has found that estrogen levels do not differ significantly between symptomatic and asymptomatic women.^64^ Instead, they are associated with a reduced thermoneutral zone in symptomatic women, where minor temperature elevations catalyze sweating and peripheral vasodilation. Thus, if mechanisms of hypnosis cannot be explained by response expectancy, it is possible that hypnosis and hypnotic suggestions work by altering activity in the medial preoptic area of the hypothalamus responsible for regulating core body temperature.

In examining moderators of hypnosis for hot flashes, our review highlights a consistent finding that those of higher hypnotizability reported fewer hot flashes postintervention than those of lower hypnotizability. At the same time, however, participants across all levels of hypnotizability eventually benefited significantly from the intervention. This idea of hot flash reduction being a matter of *when* rather than a matter of *if* was the core finding of a recent analysis done by Alldredge and colleagues^65^ examining the modulating effects of hypnotizability as a moderator. Their findings revealed that women of medium and high hypnotizability experienced a clinically significant reduction in hot flashes (50%) by the third week of the intervention, while the same benefits were not observed in participants of low hypnotizability until the 12-week follow-up assessment. Based on the findings herein, it is important to highlight relevant implications for clinical practice. Clinicians should consider hypnosis as a first-line approach for treating hot flashes because of the apparent advantage it has on reducing hot flash frequency and severity in addition to benefits it shares with CBT for improving adjacent concerns (*e.g.,* daily interference, self-reported bother).

Clinical hypnosis has become a globally available intervention, with over 30 countries reporting offering this intervention across certified clinical psychologists, physicians, social workers, and health care providers.^66^ However, clinical hypnosis is still widely underutilized despite this. Limited accessibility to practitioners offering hypnotherapy, health care costs, and time constraints are potential barriers that might contribute to this underutilization.^67^ In order to use clinical hypnosis widely to meet the pressing need of offering an effective nonpharmacological treatment for hot flashes, training in hypnosis needs to be improved and innovative methods of delivery need to be tested and disseminated. Clinicians would benefit from the availability of a fundamental training course in clinical hypnosis that focuses on the treatment of hot flashes. Accessibility of this training can be increased *via* online delivery and eligibility for continuing education hours. To our knowledge, no such resource exists and currently available training programs in clinical hypnosis are time consuming, infrequent, and do not typically focus on treating hot flashes. In addition to improved training, the accessibility of clinical hypnosis can be improved through wider dissemination of remotely delivered hypnotherapy.^67^ For instance, innovative delivery methods such as smartphone apps and hypnosis teletherapy can improve access to clinical hypnosis by addressing financial, time, or limited in-person treatment availability.

This review also carries several implications for research. Looking forward, future research can help fill some of the present gaps in the literature and examine innovative ways of delivering the hypnosis intervention. In addition to the need for additional replications of impressive findings with clinical hypnosis, a study using neuroimaging technology should be conducted to examine the neurophysiology of clinical hypnosis for hot flashes. This will help to provide a clearer understanding of the mechanisms of hypnosis and can test our speculative hypotheses around altered activity in the medial preoptic area of the hypothalamus.

Another avenue of future research, aimed at improving accessibility of hypnotherapy, should involve rigorous examination of a smartphone app-delivered hypnosis treatment for hot flashes. This would inform and improve dissemination of clinical hypnosis for hot flashes and researchers can turn to the already-developed Evia app by Mindset Health for testing. Finally, a comprehensive meta-analysis is warranted to synthesize the disparate strands of evidence and provide a broad and cohesive understanding of the effectiveness of nonpharmacological interventions for hot flashes, thus driving informed clinical decisions and patient-centered care. This suggests the importance of tailoring interventions to individual characteristics, a topic that warrants further exploration.

## Limitations

Future researchers interested in conducting follow-up studies on the use of CBT or clinical hypnosis for treating hot flashes and other postmenopausal symptoms are advised to take the following limitations into consideration when interpreting the findings of the present review. The current study did not include gray literature, ongoing studies, or studies published in non-English languages, which limited the range of findings included in the synthesis of findings included in this discussion. Additionally, given the limited number of randomized clinical trials available for clinical hypnosis and the heterogeneity across different outcome measures used to assess hot flashes across both treatment interventions, the authors were unable to provide a systematic analysis of the methodological quality, potential biases of the literature, or a meta-analyses of treatment effect size. After more evidence-based literature on hypnotherapy for hot flashes is provided, further directions would be a meta-analysis to contribute to the collective understanding of how to best advance research on this topic.

## Conclusions

Our scoping review has discerned critical insights into the differential effects of CBT and clinical hypnosis in the treatment of hot flashes. Despite both interventions being recognized and recommended by the North American Menopause Society, our analysis reveals that clinical hypnosis outperformed CBT in reducing the frequency and severity of hot flashes. Clinical hypnosis interventions have been shown to achieve clinically significant reduction in frequency and severity of vasomotor symptoms, and CBT can improve bother and daily interference of vasomotor symptoms but does not impact hot flashes. This finding bears important implications for nonpharmacological treatment of hot flashes. While CBT may aid in managing the stress and cognitive response to hot flashes, it does not effectively reduce their occurrence. In contrast, clinical hypnosis demonstrates a robust effect across single-arm trials and RCTs, marking the only nonpharmacological behavioral intervention that has demonstrated efficacy in directly influencing frequency and severity of hot flashes accompanied by the same ancillary benefits of stress reduction.

In real-world practice, if a patient is interested in both psychological and somatic benefit (reduction in hot flashes) clinical hypnosis should be recommended over CBT. Alternatively, if a patient is only interested in management of stress or bother/daily interference, CBT is a viable treatment option to address those psychological concerns. In guiding future clinical practice, clinicians should consider clinical hypnosis as a primary behavioral treatment modality for hot flashes. Future research should focus on examining the mechanisms of clinical hypnosis *via* neuroimaging studies and app-delivered modalities should be tested and compared with conventional (*i.e.,* one-on-one sessions with a clinician) delivery.

## Abbreviation Used

BDI: Beck Depression Inventory
CBT: Cognitive Behavioral Therapy
HAM-A: Hamilton Anxiety Scale
HF: Hot Flashes
HFRS: Hot Flash Rating Scale
HFRDIS: Hot Flash Related Daily Interference Scale
HT: Hormone Therapy
NS: Night Sweats
PRISMA-ScR: Preferred Reporting Items for Systematic Reviews and Meta-Analyses extension for Scoping Reviews
QLQ: Quality of Life Questionnaire
RCT: Randomized Clinical Trial
SAQ: Sexual Activity Questionnaire
